# Bacterial symbionts of the precious coral *Corallium rubrum* are differentially distributed across colony‐specific compartments and differ among colormorphs

**DOI:** 10.1111/1758-2229.13236

**Published:** 2024-03-05

**Authors:** Jeroen A. J. M. van de Water, Denis Allemand, Christine Ferrier‐Pagès

**Affiliations:** ^1^ Unité de Recherche sur la Biologie des Coraux Précieux CSM – CHANEL Centre Scientifique de Monaco Monaco Principality of Monaco; ^2^ Coral Ecophysiology Team, Department of Marine Biology Centre Scientifique de Monaco Monaco Principality of Monaco; ^3^ Department of Estuarine & Delta Systems Royal Netherlands Institute for Sea Research Yerseke The Netherlands; ^4^ Centre Scientifique de Monaco Monaco Principality of Monaco

## Abstract

Corals engage in symbioses with micro‐organisms that provide nutrients and protect the host. Where the prokaryotic microbes perform their symbiotic functions within a coral is, however, poorly understood. Here, we studied the tissue‐specific microbiota of the coral *Corallium rubrum* by dissecting its tissues from the skeleton and separating the white polyps from the red‐coloured coenenchyme, followed by *16S rRNA* gene metabarcoding of the three fractions. Dissection was facilitated by incubating coral fragments in RNAlater, which caused tissues to detach from the skeleton. Our results show compartmentalisation of the microbiota. Specifically, *Endozoicomonas*, Parcubacteria and a Gammaproteobacteria were primarily located in polyps, whereas Nitrincolaceae and one *Spirochaeta* phylotype were found mainly in the coenenchyme. The skeleton‐associated microbiota was distinct from the microbiota in the tissues. Given the difference in tissue colour and microbiota of the polyps and coenenchyme, we analysed the microbiota of three colormorphs of *C. rubrum* (red, pink, white), finding that the main difference was a very low abundance of *Spirochaeta* in white colormorphs. While the functions of *C. rubrum*'s symbionts are unknown, their localisation within the colony suggests that microhabitats exist, and the presence of *Spirochaeta* appears to be linked to the colour of *C. rubrum*.

## Graphical abstract

The distribution and functions of the symbiotic microbes of the precious coral *Corallium rubrum* are unknown. We optimised coral dissection protocols by using RNAlater, and employed *16S rRNA* metabarcoding to characterise the microbiota in different compartments (polyps, coenenchyme, skeleton) and in three colormorphs (red, pink, white). We found compartment‐specific localisation of the symbionts, and *Spirochaeta* to be nearly absent in the polyps and white colormorphs, suggesting a link between this symbiont and *C. rubrum*'s colour.

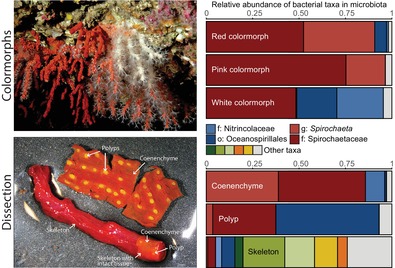

## INTRODUCTION

The microbiota of corals is generally believed to play an important role in the health of the coral holobiont, which comprises the host with its symbiotic microbial partners. These microbes may provide essential nutrients to the hosts, help with food digestion, increase stress resilience and have anti‐pathogen properties (Peixoto et al., 2017). While differences in microbiota composition have been observed between the coral mucus, tissues and skeleton (e.g., Apprill et al., 2016; Li et al., 2014; Palladino et al., 2022; Pollock et al., 2018; Sweet et al., 2011), little is known about where symbiotic microbes are located and perform their functions within the tissues of the coral, or whether tissue‐specific microhabitats exist.

Several studies have used fluorescence in situ hybridisation (FISH) to localise bacteria in coral tissues, finding mostly bacterial aggregates (Bayer et al., 2013; La Rivière et al., 2016; van de Water et al., 2015; Wada et al., 2019, 2022) located between or within tissue layers. The composition of these ‘coral‐associated microbial aggregates’ (CAMA; Wada et al., 2019) has been studied only in a few cases, and these bacteria were found to belong primarily to the genus *Endozoicomonas* (Bayer et al., 2013; Wada et al., 2022), one of the most common bacterial symbionts of corals and also of other marine invertebrates.

Methods like FISH use taxon‐specific probes, requiring prior knowledge of the microbiome. Besides, the signal needs to be sufficiently strong for detection, likely explaining why most CAMAs are found and not more sparsely distributed microbes. Using sequencing technologies, such as *16S rRNA* gene metabarcoding, the full microbial community can be profiled, including the detection of low abundant microbes, but specific tissues need to be isolated first. This is particularly a challenge because of the hard calcium carbonate skeleton of most corals studied to date, which complicates the possibility of dissecting tissues.

Ainsworth et al. (2015) approached this issue from a histology perspective on decalcified corals embedded in paraffin and using stainless steel tissue biopsy cores to obtain biopsies or laser‐capture microdissection (LCM) techniques, both in combination with *16S rRNA* gene metabarcoding. They found that certain microbial taxa specifically co‐localised with the symbiotic algal partners of these reef‐building corals or in the tissues where these algae reside. More recently, they improved biopsy‐based approaches for the analysis of the microbiota in coral holobiont microhabitats on fixed samples (Bergman et al., 2022). Wada et al. (2022) also used LCM to further characterise the CAMA communities using metagenomics in combination with stable isotope analyses, revealing a role for *Endozoicomonas* in phosphate cycling. Studies on fixed specimens are somewhat sub‐optimal though, as the DNA extracted from fixed tissues is often degraded. Compartmentalisation of microbial communities has also been studied in corals with accessible larger polyps (Agostini et al., 2012; Engelen et al., 2018), where the gut content/gastric fluid was sampled with a syringe, the mucus layer was swapped and the whole polyp was dissected. These studies showed distinct communities present in the gastric cavity of corals, but similar studies on corals with small polyps are unfeasible. The spatial microbial communities of the soft‐bodied sea anemone *Nematostella vectensis* have also been studied but through dissection and *16S rRNA* gene amplicon sequencing (Bonacolta et al., 2021), showing that the microhabitat of its Spirochaetes symbionts was in the capitulum (pharynx and tentacles) and not elsewhere in the animal's body.

**FIGURE 1:**
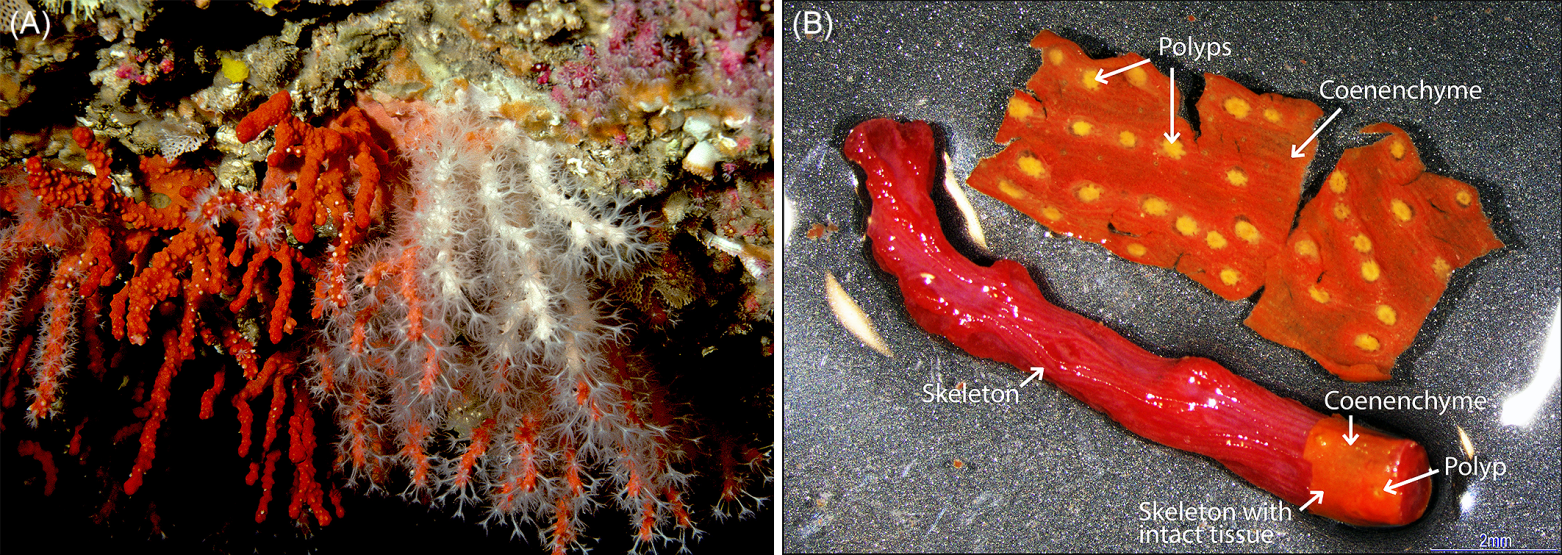
The precious red coral *Corallium rubrum*. (A) Photo showing the common red and very rare white colormorphs of *C. rubrum* in its coralligenous habitat in the Mediterranean Sea in the Parc National des Calanques near Marseille. Also clearly visible are the white polyps (fully extended, and partially and fully retracted) contrasting with the red coenenchyme tissues. Photo by © Jean‐Georges Harmelin. (B) Dissection of *C. rubrum*. Overnight incubation of the coral fragment in RNAlater at 4°C, causes tissues to detach from the skeleton. Tissues were removed from the coral skeleton, and polyps (now easily accessible from the calicodermal/skeleton side) were dissected from the coenenchyme. Bottom right corner: a part of the skeleton with the tissues still present.

Our model species is the precious coral *Corallium rubrum* (Figure 1A), which is an octocoral endemic to the Mediterranean Sea, whose red and hard calcium carbonate skeleton has been used in arts and jewellery since Antiquity. This coral possesses a unique microbiota, stable in space and time, dominated also by bacteria belonging to the Spirochaetes (van de Water et al., 2016, 2018). We, therefore, aimed to investigate whether specific microhabitats exist in the *C. rubrum* holobiont using *16S rRNA* gene metabarcoding.

## EXPERIMENTAL PROCEDURES

### Biological material and sample processing

Fragments of ~5 cm long of *C. rubrum* (*n* = 5) were collected in November 2018 at 35 m depth in the Parc National des Calanques (France). Fragments were stored in RNAlater (Thermo Fischer Scientific) at 4°C overnight. We found that such an incubation would cause near complete detachment of the tissues from the skeleton. The next day, tissues were removed from the skeleton (Figure 1B), and the tissues were further dissected into a polyp fraction and a coenenchyme fraction using sterile forceps and scalpels. Tissue remnants on the skeleton were removed using sterilised interdental brushes to limit contamination of the skeleton fraction. Each fraction was stored in RNAlater at 4°C until further processing.

Colormorphs of *C. rubrum*—red (*n* = 4), pink/light red (*n* = 4), and the very rare white colormorph (*n* = 4) (Figure 1A)—had been collected in the Parc des Calanques in 2015 and maintained for 3 years in open‐flow aquaria at 15°C in the dark at the Centre Scientifique de Monaco. [Of note, two of the four coral fragments (CrWhite1 and CrWhite4) that were initially sampled as white colormorphs, changed to pink colormorphs during our study, and the data of these samples were not included for further analysis.] A fragment of approximately 1 cm was cut from the colony and preserved in RNAlater at 4°C until further processing. Four litres of seawater from the aquaria were filtered on a 0.22 μm Whatman Nuclepore Track‐Etched filter (Sigma‐Aldrich), and the filter was stored in RNAlater at 4°C. All collections were conducted with the permission of the Direction Inter‐régionale de la Mer Mediterranée (France).

### DNA extraction and sequencing

DNA was extracted using the DNeasy PowerBiofilm kit (Qiagen) according to the manufacturer's protocol, including eight negative controls, with the exception that the bead beating was performed at 30 Hz for 10 min (5× 2 min) using a CryoMill (Retsch, Haan, Germany) at room temperature. DNA extracted from the dissected fractions (polyp, coenenchyme and skeleton [crushed to powder using a mortar and pestle]) were shipped to STAB‐VIDA (Portugal) for sequencing library construction following the protocol in the ‘16S Metagenomic Sequencing Library Preparation Guide’ (Illumina, 2013) using the 27F (5′‐AGAGTTTGATCCTGGCTCAG‐3′) and 338R (5′‐TGCTGCCTCCCGTAGGAGT‐3′) primer set that targets the V1–V2 regions of the *16S rRNA* gene. DNA extracted from the *C. rubrum* colormorphs was shipped to MR DNA (Shallowater, TX, USA) for sequencing library construction using the 27F (5′‐AGAGTTTGATCCTGGCTCAG‐3′) and 519R (5′‐GTATTACCGCGGCTGCTG‐3′) primers that target the V1–V3 regions of the *16S rRNA* gene. Libraries were paired‐end (2 × 300 bp) sequenced on the Illumina MiSeq platform with V3 chemistry.

### 16S rRNA gene amplicon sequence data processing and analysis

The two different *16S rRNA* gene amplicon datasets were, because of the different but overlapping primer sets, separately processed using the UNOISE3 pipeline as implemented in the USEARCH package (version 11; https://www.drive5.com/usearch/) (Edgar, 2010). The raw forward (R1) and reverse (R2) sequence fastq files contained a total of 2,872,382 reads (localisation; range 328–124,427 reads per sample) and 2,223,604 reads (colormorphs; range 32,843–109,576 reads per sample) with average Q20 scores of 73.53% and 86.59%, and Q30 scores of 61.27% and 73.79%, respectively. R1 and R2 paired reads were merged using ‐fastq_mergepairs. Primer sequences were trimmed using ‐fastx_truncate and reads were quality filtered with the ‐fastq_filter script, generating filtered fasta files containing 1,089,943 reads with an average length of 307 bp (localisation) and 432,286 reads with an average length of 477 bp (colormorph). Unique sequences were identified using the ‐fastx_uniques script followed by denoising of the sequence dataset with the UNOISE3 algorithm, obtaining respectively 692 (localisation) and 1141 (colormoph) denoised sequences or ‘zero‐radius OTUs’ (zOTU, Operational Taxonomic Unit). The –usearch_global script was then used to generate OTU tables at the 99% similarity level—this level was chosen to minimise data loss due to a few ambiguous base calls in a read while preserving all zOTUs (i.e., no zOTUs were merged due to sequence similarity at the 99% level). The localisation OTU table contained 692 OTUs with an average of 77,964 reads per sample (localisation; range 11,823–100,801 reads), but skeleton samples 1 and 2 were discarded due to failed sequencing (64 and 44 reads, respectively) and negative controls contained 212–386 reads. The colormorph OTU table contained 1141 OTUs with an average of 39,029 reads per sample (colormorphs; range 18,314–61,890 reads), but white colormorph samples 1 and 4 were discarded as the colour of the mother colony had changed from white to pink. The taxonomy was assigned to each OTU based on the SILVA database (release v138) (Quast et al., 2013) using the ‐sintax algorithm. The OTU table was converted to the HDF5 .biom format and taxonomic assignment metadata was added. The unfiltered OTU tables, sample metadata and representative sequences of each OTU are provided in the Supporting Information.

To control for potential contaminants in the sequencing datasets as an additional quality control step, two different methods implemented in the R‐package *decontam* (Davis et al., 2018) were used. The localisation dataset contained negative extraction controls, and the *isContaminant* function was used to identify and filter out 43 potential contaminant OTUs. As negative extraction controls had not been included in the colormorph dataset, the ‘Frequency’ method, a statistical approach, was employed instead. Six OTUs were identified as potential contaminants and removed prior to statistical data analysis. OTUs identified as chloroplasts or mitochondria were also removed from both datasets.

To investigate the coral‐associated prokaryotic communities, the Compositional Data (CoDa) approach was used (Gloor et al., 2017) to account for the large number of zero count values that are due to under‐reporting or limited sampling (e.g., insufficient or unequal sequencing depth). In short, zero counts were imputed using a Bayesian Multiplicative Replacement procedure (Bayes‐LaPlace method; R‐package *zCompositions*; Palarea‐Albaladejo & Martín‐Fernández, 2015) under the assumption of a Dirichlet prior distribution. Then, a centred log‐ratio (clr) transformation was applied and the Euclidean distances between samples were calculated to obtain an Aitchison Distance Matrix. Next, Permutational Multivariate Analysis of Variance (perMANOVA; *adonis* function) in combination with analyses of group dispersions (*betadisper* function) as implemented in the R‐package *vegan* (Oksanen et al., 2018) and pairwise perMANOVA as implemented in the R‐package *metagMisc* (Mikryukov, 2017) were used to assess differences in beta diversity between species and treatments, and outcomes were visually represented in distance‐based Redundancy Analysis (dbRDA) ordination plots. Differential abundance analyses were performed using the Analysis of Compositions of Microbiomes with Bias Correction (*ancombc2* function; ANCOM‐BC [Lin & Peddada, 2020]). The *phyloseq* package (McMurdie & Holmes, 2013) integrated in R was used to generate relative abundance plots, and heatmaps of differentially abundant OTUs.

## RESULTS & DISCUSSION

To this end, we used a novel, relatively rapid, approach to remove nearly intact tissues from the hard calcium carbonate skeleton of the precious coral *C. rubrum* and dissect the white polyps from the red‐coloured coenenchyme (Figure 1A,B). We found that an overnight incubation of coral fragments in the DNA/RNA‐preserving buffer RNALater resulted in the detachment of the tissue layers from the skeleton, facilitating the separation of the tissues from the skeleton. Further dissection of the polyps and coenenchyme from the detached tissues and subsequent DNA extractions with the DNeasy PowerBiofilm kit (Qiagen) allowed us to study the composition of the bacterial communities in these three main compartments—polyp, coenenchyma and skeleton—using metabarcoding of the V1–V2 regions *16S rRNA* gene. The amplicon sequencing data was processed with the USEARCH pipeline as per (Edgar, 2010) and statistical analyses were performed following the Compositional Data (CoDa) approach (Gloor et al., 2017). The outcomes of statistical analyses can be found in the Supporting Information.

**FIGURE 2:**
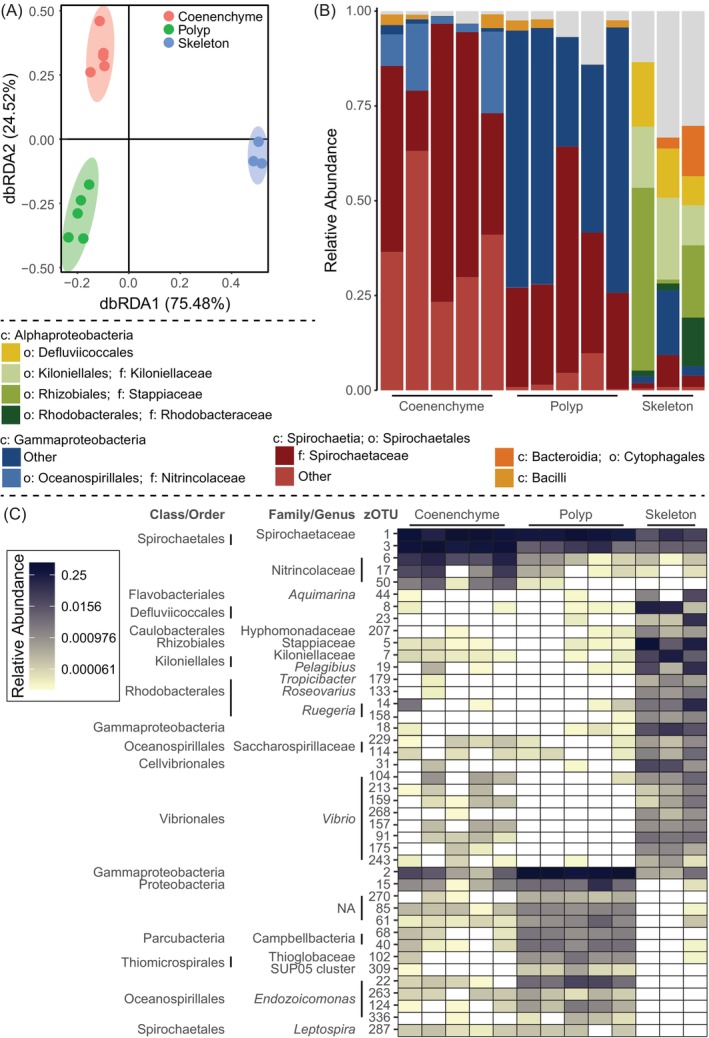
Microbial community of tissue fractions and skeleton of *Corallium rubrum*. (A) Beta diversity of the prokaryotic communities associated with the polyps, coenenchyma and skeleton. (B) Composition of the prokaryotic communities harboured within the polyps, coenenchyma and skeleton. (C) Bacterial phylotypes (zOTUs) that are differentially abundant between the three fractions as analysed using *ANCOM‐BC*. The results of the statistical analyses are provided in the Supporting Information.

Our results show that the microbiota of the red coral is compartmentalised as there are clear differences in the diversity of the microbial communities between the compartments (Figure 2A; *p* = 0.0198 for all pairwise comparisons). This is also reflected in the community composition (Figure 2B). Differential abundance analyses using *ANCOM‐BC2* (Lin & Peddada, 2020) identified especially a limited number of phylotypes (1) higher abundant in polyps than coenenchyme (particularly *Endozoicomonas*, Parcubacteria, Thiomicrospirales and a highly abundant Gammaproteobacteria [zOTU2]); (2) higher abundant on coenenchyme than polyps (Nitrincolaceae and one Spirochaetales [zOTU3]), whereas the relative abundance of the main symbiont zOTU1 (Spirochaetaceae) did not differ between the two tissue compartments (Figure 2C). Interestingly, these phylotypes all matched 100% with previously identified members of the core microbiome of *C. rubrum* (van de Water et al., 2016, 2018) (Table E in the Supporting Information). The skeleton‐associated microbial community (on and/or in [i.e., endolithic] the skeleton) of *C. rubrum* was highly distinct from the microbiota in the tissues, and was primarily composed of members from various orders within the Alphaproteobacteria, consistent with previous reports on tropical reef‐building corals (Cárdenas et al., 2022; Liu et al., 2022; Yang et al., 2020), including Rhizobiales, Kiloniellales and Rhodobacterales. Other common skeleton‐associated microbes belonged to Gammaproteobacteria, of which the Saccharospirillaceae, Cellvibrionales, and a diverse community of *Vibrio* phylotypes were the most characteristic (Figure 2C). Although some of these phylotypes had previously been found at very low abundances in the microbiota of *C. rubrum* (van de Water et al., 2016, 2018), this is the first characterisation of the composition of its skeleton‐associated prokaryotic community. Most strikingly may be the observation that *Vibrio* bacteria (possibly *V. coralliirubri* [Poli et al., 2018]) are mostly associated with the skeleton of this coral, raising the question of whether the skeleton may provide microhabitats for potential opportunistic pathogens, as *Vibrio* spp. have been implicated in various coral diseases (e.g., Arotsker et al., 2009; Sussman et al., 2008; Ushijima et al., 2014, 2020). Where these microbes are exactly located—inside the skeleton along the organic matrix (i.e., an endolithic community) or on the surface of the skeleton—remains to be studied. While the functions of these microbes are still unknown, our results show that the main microbial symbionts of *C. rubrum* are hosted in different parts of the holobiont.

**FIGURE 3:**
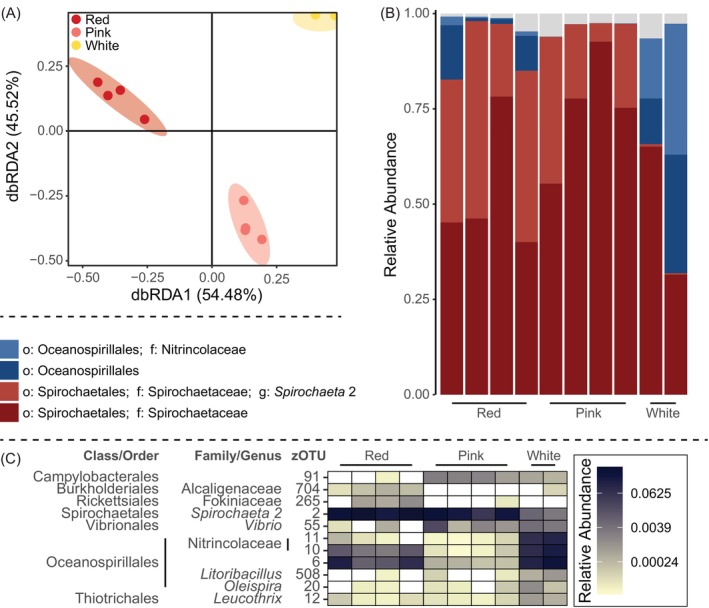
Microbial community of colormorphs of *Corallium rubrum*. (A) Beta diversity of the prokaryotic communities associated with the red, pink and white colormorphs. (B) Composition of the prokaryotic communities harboured within the different colormorphs. (C) Bacterial phylotypes (zOTUs) that are differentially abundant between the red, pink and white colormorphs as analysed using *ANCOM‐BC*. The results of the statistical analyses are provided in the Supporting Information.

Given the differences in colour of the tissues, we further studied whether some of these microbes may be characteristic of the red colour of the tissues of *C. rubrum* by investigating the microbiota of normal, red‐coloured *C. rubrum* and that of pink/light red and the rare white colormorphs of this species that had been kept in aquaria for over 3 years prior to sampling (Figure 1A). The procedures used were largely the same, except that the metabarcoding was performed on the slightly larger amplicons of the V1–V3 regions of the *16S rRNA* gene. Results show significant differences in the diversity of the microbial communities associated with the colormorphs (Figure 3A; *p* = 0.0004). But this was mostly the result of a near absence of zOTU2 (*Spirochaeta*) and zOTU80 (*Leptospira*) and concomitantly higher relative levels of Oceanospirillales and Nitrincolacaea OTUs in white colormorphs in comparison with the red and pink *C. rubrum* (Figure 3B,C). However, the same Oceanospirillales and Nitrincolacaea OTUs were less abundant in pink than in red colormorphs, whereas the opposite was the case for Campylobacterales (Figure 3C). Other low‐abundant OTUs were also found to be differentially abundant between colormorphs but did not match with any previously identified phylotypes from natural samples and are therefore not further discussed here. The main zOTUs, however, matched the previously identified core microbiome members (van de Water et al., 2016, 2018), allowing us to match the OTUs in this study with their likely counterparts from the localisation study using the BLASTn algorithm (Altschul et al., 1990) (Table E in the Supporting Information).

The specific localisation of the bacterial symbionts of *C. rubrum* raises the question of what function they perform in their microhabitats. Previously, *Endozoicomonas* have been implicated in nitrogen and sulfur cycling, amino acid synthesis as well as the breakdown of complex molecules and microbiome regulation (Neave et al., 2016). Their localisation in the red coral polyps would support the hypothesis that they aid with the digestion of prey and protect against potentially harmful microbes entering through the mouth. The functions of the other symbionts of *C. rubrum* belonging to the Gammaproteobacteria, Parcubacteria and Spirochaetes are still unknown.

However, the observations that (1) white colormorphs of *C. rubrum* have very low abundances of *Spirochaeta* (zOTU2) compared with red colormorphs, and that (2) the matching phylotype from the localisation study (zOTU3) is present in the red coenenchyme tissues but not in the white polyps, suggest a link between the red colour of the coral colony and the presence of this *Spirochaeta*. The red colour, which determines the commercial value of red coral, originates from carotenoids incorporated in the tissues and skeleton (Cvejic et al., 2007). The exact origin of the carotenoids remains to be identified, but we believe (based on yet unpublished genomics data—P. Ganot) that the coral obtains carotenoids or intermediates from the carotenoid synthesis pathway from external sources, likely of microbial nature. In contrast to other Spirochaetales genera, bacteria from the genus *Spirochaeta* have previously been shown to produce carotenoids (Greenberg & Canale‐Parola, 1975). Taken together, we hypothesise that *Spirochaeta* may play a role in the pigmentation of *C. rubrum*. Metagenomics approaches will, however, be needed to provide further valuable insights into a microbial contribution, and that of *Spirochaeta* in particular, to *C. rubrum*'s colour.

Dissection approaches, as presented here, may open possibilities to obtain tissue‐specific metagenomes and identify the functions of the microbial symbionts located there, or even tissue‐specific transcriptome profiling. However, it would also be interesting to advance spatial ‘omics technologies to further investigate where the various microbial symbionts are located in the coral tissues and simultaneously study which functions they perform in their respective microhabitats.

## AUTHOR CONTRIBUTIONS

**Jeroen A. J. M. van de Water:** Conceptualization (lead); data curation (lead); formal analysis (lead); funding acquisition (supporting); investigation (lead); methodology (lead); visualization (lead); writing – original draft (lead); writing – review and editing (lead). **Denis Allemand:** Funding acquisition (lead); writing – review and editing (supporting). **Christine Ferrier‐Pagès:** Funding acquisition (supporting); writing – review and editing (supporting).

## CONFLICT OF INTEREST STATEMENT

The authors declare no conflict of interest.

## Supporting information


**Data S1:** Supporting Information.

## Data Availability

The raw sequencing data is available via the NCBI Sequence Read Archive under accession numbers PRJNA983747 and PRJNA983964. Processed data (OTU table, metadata and OUT sequences) are in the Supporting Information.
